# New Appliances for Hospital Use

**Published:** 1907-06-29

**Authors:** 


### NEW APPLIANCES FOR HOSPITAL USE.

### A NEW HOT-WATER HEATING APPLIANCE.

A new appliance designed for the purpose of heating by hot-water pipes or radiators from an ordinary open fireplace is supplied by Mr. J. D. Prior, Empire Works, Holliday Street, Birmingham. A series of oblong copper tubes is placed in a vertical position at the back of the fireplace in such a manner as to receive the heat, these tubes being connected with the flow and return pipes of the circulatory system of hot-water heating. The tubes are constructed so as to give elasticity for expansion, this, together with their shape and weight, being such that they resist injury by blows from a poker. For greater strength they are made seamless. Offering a cheap and ready means of installing radiators or pipes for additional heating in wards otherwise unprovided with sufficient warmth, this device should prove very useful. It is made in two sizes capable of heating from 60 to 80 feet of heating surface in the radiators. It is listed at 65s. to 75s., and has been awarded two first prize medals by the Royal Sanitary Institute at the Bristol Health Exhibition this year.

### TO BRIGHTEN ASPHALTE FLOORING.

Asphalte flooring can be stained by using a mixture of blacklead and turpentine in the proportions found to give the required depth of tint to suit individual taste, the colour being a grey shade, varying from a delicate hue to practically black. This has been done to the corridor floors at several institutions, and looks very neat. It is painted on with a brush or mop, and after being once done merely requires a light treatment in the same way about once a fortnight, the labour being practically no more than scrubbing the floors for cleaning. The cost is trifling.

### A NEW DEVICE FOR CLEANSING FOUL LINEN.

A new machine for dealing with fouled and offensive linen in hospital laundries is now being made by Messrs. Entwisle and Gass, Ltd., of Bolton, Lancs. This machine avoids the use of steeping tanks or the handling of the linen except in the process of loading the machine, and consists essentially in the attachment of an automatic flushing device to the external cage of the washer, alternately flooding and emptying the inner compartment by syphonic action. The linen is placed in the machine exactly as received at the laundray, a little soap powder and possibly disinfectant being added. The lid is then closed, and the machine, of the ordinary rotary cage type, set in motion. The flooding cistern then gradually fills the cage until the level of the emptying syphon is reached, the whole of the liquid contents being rapidly discharged, sweeping out all sediment at the same time. The first flush usually takes away most of the foul matter, and by the time the third flush has taken place the linen is generally ready for removal. The operation thus described is completely automatic, and an attachment is added to prevent the flushing arrangement being left in action when the machine is stopped, thus preventing waste of water. This foul-linen cleansing arrangement can be easily applied to existing machines, and is certainly a great improvement over present methods. It has been applied at several institutions.

